# Inhibition of heat shock protein 27 phosphorylation promotes sensitivity to 5-fluorouracil in colorectal cancer cells

**DOI:** 10.3892/ol.2014.2580

**Published:** 2014-10-01

**Authors:** ATSUSHI MATSUNAGA, YOSHIYUKI ISHII, MASASHI TSURUTA, KOJI OKABAYASHI, HIROTOSHI HASEGAWA, YUKO KITAGAWA

**Affiliations:** Department of Surgery, Keio University School of Medicine, Tokyo 160-8582, Japan

**Keywords:** heat shock protein 27 (HSP27), 5-fluorouracil, phosphorylation, sensitivity, colorectal cancer

## Abstract

The aim of the present study was to investigate whether the inhibition of HSP27 phosphorylation, which affects certain cellular functions, modulates sensitivity to 5-fluorouracil (5-FU) in colorectal cancer cells. Exposure to 5-FU in HCT116 and HCT15 cells expressing high levels of HSP27 with a low 5-FU sensitivity caused a minimal change in HSP27 expression, but induced the upregulation of HSP27 phosphorylation, particularly at Ser78. By contrast, exposure to 5-FU in HT29 cells expressing a low level of HSP27 with a high 5-FU sensitivity marginally increased HSP27 expression, with minimal phosphorylation. Treatment with a selective inhibitor, p38 mitogen-activated protein kinase (MAPK; SB203580), caused the dose-dependent suppression of HSP27 phosphorylation, which was upregulated by 5-FU, reducing the half maximal inhibitory concentration values of 5-FU in the HCT116 and HCT15 cells. However, treatment with SB203580 exhibited no significant effect on cell growth or survival. In conclusion, this study indicated that the inhibition of HSP27 phosphorylation by a selective inhibitor of p38 MAPK promotes 5-FU sensitivity without causing cytotoxicity in colorectal cancer cells.

## Introduction

5-Fluorouracil (5-FU) is a key chemotherapy drug for colorectal cancer, and therefore, the inherent or acquired resistance to 5-FU is a serious problem. A number of studies have reported the association between the response to or toxicity of the drug and 5-FU metabolism-associated factors, including thymidylate synthase, dihydropyrimidine dehydrogenase, folate cofactors and orotate phosphoribosyltransferase (1–4). However, at present no reliable biomarkers for the sensitivity or resistance to 5-FU chemotherapy have been identified.

Mammalian heat shock proteins (HSPs) such as HSP90, HSP70, HSP60 and small HSPs (15–30 kDa), including HSP27, are present in numerous organs and are considered as molecular chaperones in protein-protein interactions, including folding, unfolding and assembly, as well as anti-apoptotic proteins and contributors to cell survival (5,6). Numerous studies have reported that HSP27 expression contributes to the malignant properties of cancer cells, including the resistance to treatment, tumorigenicity and the inhibition of apoptosis (7–11). In colorectal cancer, certain studies have reported that the expression of HSP27 is involved in doxorubicin or irinotecan resistance *in vitro* (12,13), and recent clinicopathological studies have revealed HSP27 to be a prognostic marker (14–16). Our previous studies also indicated that the protein levels of HSP27 expression contributed to the degree of resistance to 5-FU in studies performed *in vitro* and *in vivo* using a xenograft model (17,18).

A variety of stimuli induce the phosphorylation of serine residues 15, 78 and 82 in HSP27 using various protein kinases, such as mitogen-activated protein kinase (MAPK) activated protein kinase 2 (MAPKAPK-2), via p38 MAPK (19–24). This post-translational modification affects a number of the cellular functions of HSP27. As a consequence of the functional importance of HSP27 phosphorylation, aberrant HSP27 phosphorylation has been linked to several clinical conditions, including cancer progression and the malignant behavior of a number of cancer types (19). Pathogenic conditions associated with aberrant HSP27 phosphorylation, as well as potential therapeutic strategies aimed at modulating HSP27 phosphorylation, are expected to be developed in the future.

The present study aimed to clarify whether HSP27 expression or phosphorylation was modulated by 5-FU exposure and to investigate whether the inhibition of HSP27 phosphorylation by a specific kinase inhibitor affected the sensitivity of colorectal cancer cells to 5-FU.

## Materials and methods

### Drug, cell lines and cell culture conditions

The anticancer drug 5-FU was purchased from Kyowa Hakko Bio Co., Ltd. (Tokyo, Japan). A selective p38 MAPK inhibitor, SB203580 (20), was purchased from Promega Corporation (Madison, WI, USA). The human colon cancer cell lines, HCT116, HCT15 and HT29, were obtained from the American Type Culture Collection (Manassas, VA, USA). The cells were grown in RPMI-1640 medium (Gibco-BRL, Carlsbad, CA, USA). Each culture was supplemented with 10% heat-inactivated fetal bovine serum (FBS; CSL Ltd., Melbourne, Australia) and 1% penicillin/streptomycin (1 ml) in a humidified 5% CO_2_ incubator at 37°C. Prior to the experiments, the cells were incubated without FBS for 24 h.

### Western blot analysis for HSP27 and phosphorylated HSP27

Subconfluent cells were exposed or not exposed to 5-FU (1.28 μg/ml) with SB203580 treatment (0, 1 or 10 μM) in culture medium for 48 h. The total cell lysates were then extracted using lysis buffer [20 mM Tris/HCL (pH 7.5), 150 mM NaCl, 1 mM EDTA, 1 mM EGTA, 1% TritonX-100, 2.5 mM sodium pyrophosphate, 50 mM NaF, 50 mM HEPES, 1 mM Na_3_VO_4_ and 2 mM phenylmethylsulfonyl fluoride; Cell Signaling Technology, Inc., Danvers, MA, USA]. The quantity of cell lysates was determined using a Bio-Rad DC Protein Assay kit (Bio-Rad, Hercules, CA, USA), and a total of 20 μg lysates were resolved in Ready Gel (Bio-Rad) and transferred to an Immuno-Blot polyvinylidene fluoride membrane (Bio-Rad). The membrane was blocked with phosphate-buffered saline (PBS; Gibco-BRL) containing 5% skimmed milk powder for 2 h at room temperature, then incubated at 4°C overnight with anti-human HSP27 mouse monoclonal antibody (1:2,000; G3.1; Lab Vision Corporation, Fremont, CA, USA), anti-human phospho-HSP27 (Ser15) rabbit polyclonal antibody (1:500; Upstate Biotechnology, Inc., Lake Placid, NY, USA), anti-human phospho-HSP27 (Ser78) mouse monoclonal antibody (1:2,000; Upstate Biotechnology, Inc.), anti-human phospho-HSP27 (Ser82) rabbit polyclonal antibody (1:1,000; Upstate Biotechnology, Inc.) or anti-human β-actin mouse monoclonal antibody (1:5,000; AC74; Sigma-Aldrich, St. Louis, MO, USA). The membranes were incubated for 30 min with a horseradish peroxidase-conjugated anti-mouse immunoglobulin G (IgG) (1:2,500; Promega Corporation) or anti-rabbit IgG (Promega Corporation). Bound complexes were detected using the ECL-Plus reagent (GE Healthcare Life Sciences, Chalfont, UK) according to the manufacturer’s instructions. Each experiment was performed in triplicate.

### Cell proliferation assay

A cell proliferation assay was performed using the 3-(4,5-dimethylthiazol-2-yl)-2,5-diphenyl bromide (MTT) assay, as described previously (17). Briefly, 5,000 cells/well in 96-well microtiter plates (Sumilon; Sumitomo Bakelite Co., Ltd., Tokyo, Japan) were incubated for 24 h and exposed to various concentrations of 5-FU with SB203580 treatment (0, 1, 10 or 50 μM) for 48 h, followed by a drug-free medium for an additional 24 h. The absorbance in the wells was measured using an NJ-2300 microplate spectrophotometer at wavelengths of 540 and 630 nm (Immuno Reader; Nalge Nunc International, Rochester, NY, USA). The inhibition rate was calculated using the following formula: Inhibition rate (%) = [1 - (mean absorbance of drug wells / mean absorbance of control wells)] × 100. The absorbance of each well was adjusted using the mean absorbance of the blank wells. 5-FU sensitivity was evaluated using the half maximal inhibitory concentration (IC_50_) value, which corresponded to the concentration of the drug required to inhibit cell growth by 50% relative to the untreated cells.

A cell count of viable cells was also performed to evaluate cell proliferation. Viable cells were counted four days after plating in triplicate dishes in various concentrations of SB203580 (0, 1, 10 or 50 μM) in RPMI-1640 containing 5% FBS.

### Statistical analysis

Data are expressed as the mean ± standard deviation. The statistical analysis was performed using Student’s t-test or the Mann-Whitney U test. P<0.05 was considered to indicate a statistically significant difference.

## Results

### Upregulation of HSP27 phosphorylation following exposure to 5-FU in colorectal cancer cells

**Figure 1:**
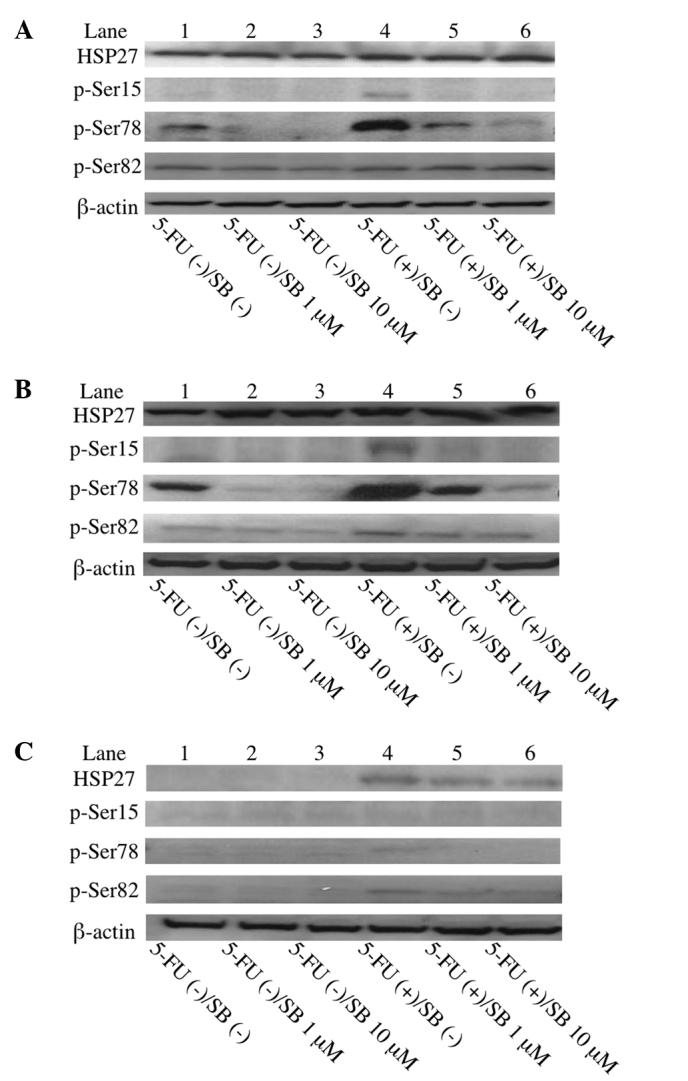
Western blot analysis of phosphorylated HSP27 expression in colorectal cancer cells. 5-FU exposure upregulated HSP27 phosphorylation, particularly Ser78, in HCT116 and HCT15 cells with high levels of HSP27 expression, but not in HT29 cells with a low level of HSP27 expression. SB203580 treatment dose-dependently inhibited HSP27 phosphorylation in HCT116 and HCT15 cells. (A) HCT116; (B) HCT15; and (C) HT29. Lanes 1–3, no 5-FU treatment; lanes 4–6, 5-FU treatment; lanes 2 and 5, treatment with 1 μM SB203580; and lanes 3 and 6, treatment with 10 μM SB203580. HSP, heat shock protein; 5-FU, 5-fluorouracil; SB, SB203580.

The effect of 5-FU exposure on HSP27 expression or phosphorylation was examined in colorectal cancer cells. Colorectal cancer cells expressing high levels of HSP27 with a low sensitivity to 5-FU (HCT116 and HCT15) and those expressing a low level of HSP27 with a high sensitivity to 5-FU (HT29) (17) were used. Although the exposure of the HCT116 and HCT15 cells to 5-FU exhibited a minimal effect on HSP27 expression (Fig. 1A and B), the exposure of the HT29 cells to 5-FU caused a marginal increase in HSP27 expression (Fig. 1C). Furthermore, exposure to 5-FU mainly upregulated the phosphorylation of HSP27 at Ser78 in the HCT116 and HCT15 cells (Fig. 1).

### Inhibition of HSP27 phosphorylation by a selective inhibitor of p38 MAPK (SB203580) in colorectal cancer cells

The effect of SB203580, a p38 MAPK selective inhibitor (20), on the phosphorylation of HSP27 was investigated. Treatment with SB203580 attenuated the phosphorylatio of HSP27, particularly at Ser78, without 5-FU exposure in the HCT116 and HCT15 cells. In addition, SB203580 dose-dependently suppressed the phosphorylation of HSP27 at Ser78, which had been upregulated following exposure to 5-FU, in the HCT116 and HCT15 cells (Fig. 1A and B). These results indicated that the stress of 5-FU exposure upregulates the phosphorylation of HSP27, particularly at Ser78, via p38 MAPK in HCT116 and HCT15 cells.

### Promotion of 5-FU sensitivity by inhibition of HSP27 phosphorylation in colorectal cancer cells

**Figure 2:**
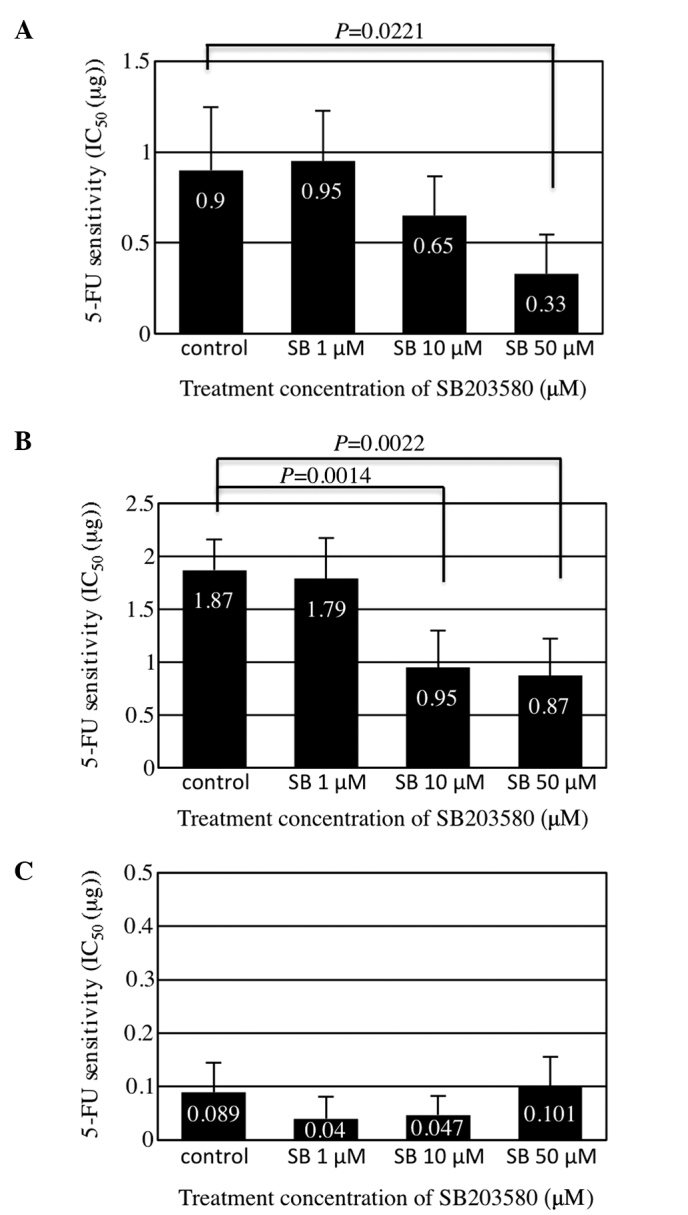
5-FU sensitivity (IC_50_) following SB203580 treatment. The HCT116 and HCT15 cells with high levels of HSP27 expression exhibited higher 5-FU IC_50_ values than the HT29 cells with a low level of HSP27 expression in the control. SB203580 treatment significantly reduced the 5-FU IC_50_ values in the HCT116 and HCT15 cells. (A) HCT116; (B) HCT15; and (C) HT29. IC_50_, half maximal inhibitory concentration; HSP, heat shock protein; 5-FU, 5-fluorouracil; SB, SB203580.

The inhibition of cell growth by 5-FU was analyzed using the MTT assay and 5-FU sensitivity was determined using the IC_50_ value. The HCT116 and HCT15 cells expressing high levels of HSP27 exhibited high IC_50_ values, while the HT29 cells expressing a low level of HSP27 exhibited a low IC_50_ value (Fig. 2), as previously reported (17). Treatment with SB203580 significantly reduced the IC_50_ values of 5-FU in the HCT116 and HCT15 cells, which exhibited high levels of HSP27 phosphorylation following 5-FU exposure (Fig. 2). These results indicated that the inhibition of HSP27 phosphorylation by SB203580 promotes sensitivity to 5-FU in colorectal cancer cells with high levels of HSP27 expression.

### Effect of SB203580, a p38 MAPK selective inhibitor, on colorectal cancer cell growth

**Figure 3:**
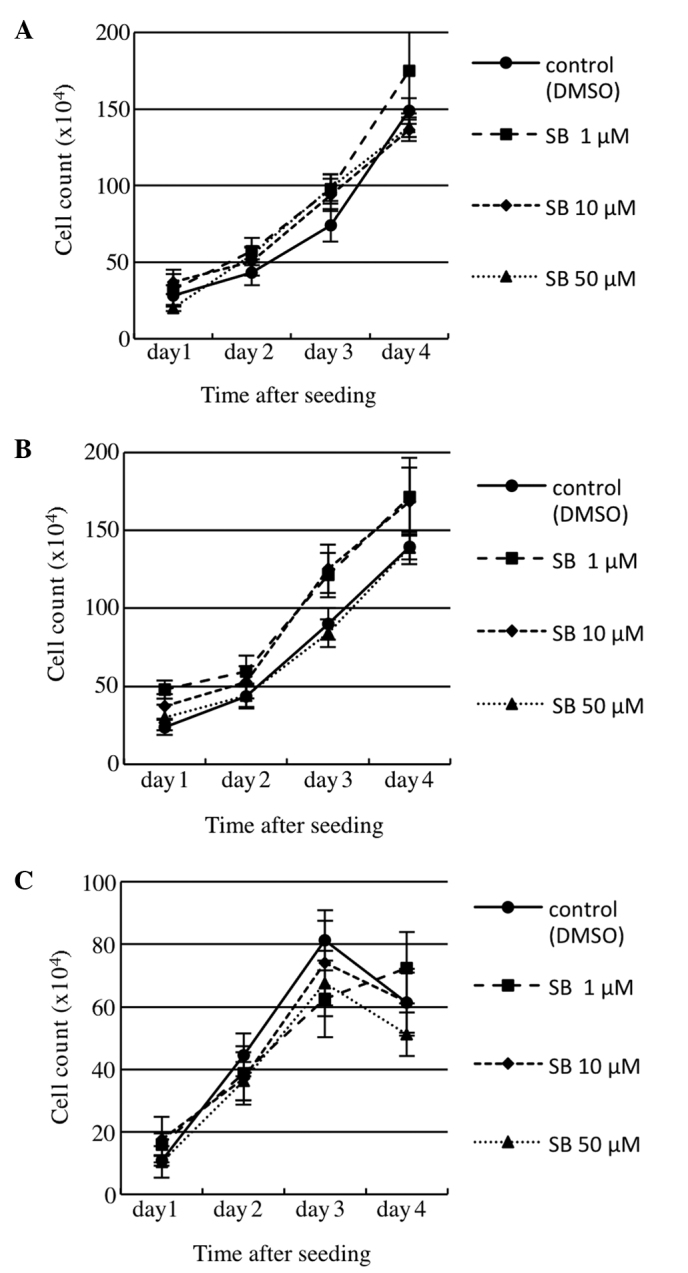
Cell growth assay following SB203580 treatment. The cells were treated with various concentrations of SB203580 (0, 1, 10 and 50 μM), and viable cells were counted for four days. No significant differences in the cell growth of the three cell lines was identified among the various SB203580 concentrations used. (A) HCT116; (B) HCT15; and (C) HT29. HSP, heat shock protein; 5-FU, 5-fluorouracil; SB, SB203580; DMSO, dimethyl sulfoxide.

To investigate the effect of SB203580 on cell growth or survival, a cell growth assay was performed using a count of viable cells. Three colorectal cancer cell lines were examined following treatment with various concentrations of SB203580 (0, 1, 10 or 50 μM) in RPMI-1640 containing 5% FBS (Fig. 3). As a result, SB203580 treatment exhibited no significant effect on cell growth or survival in the cell lines.

## Discussion

HSP27 is well-known as a stress-activated, adenosine triphosphate-independent cytoprotective chaperone that is associated with numerous functions, including the resistance to chemotherapy. HSP27 has been reported to be a clinical prognostic factor or as a resistant factor for the cytotoxic agent irinotecan in colorectal cancer studies performed *in vitro* (13–16). In addition, using *in vitro* and *in vivo* studies performed with colorectal cancer cells, we previously demonstrated that the overexpression of HSP27 reduced 5-FU sensitivity, while the suppression of HSP27 expression promoted 5-FU sensitivity. In addition, the promotion of 5-FU sensitivity by the suppression of HSP27 expression resulted in the induction of apoptosis despite the upregulation of 5-FU metabolism (17,18).

The functions of HSP27 are considered to be regulated by post-transcriptional modifications, such as phosphorylation (21,22). Human HSP27 is phosphorylated at three sites, namely, Ser15, 78 and 82 (21). These serine residues have been identified as sites for a kinase known as MAPKAPK-2 (23), and p38 MAPK is known to act as a direct kinase for MAPKAPK-2 (24), thus, p38 MAPK may phosphorylate HSP27. The phosphorylation of HSP27 promotes the dissociation of large oligomers, induces nuclear translocation and finally becomes involved in cellular protection by HSP27 (25). However, the role of HSP27 phosphorylation in chemoresistance or sensitivity remains unclear.

Recently, HSP27 has been identified as a treatment target for several cancers, and clinical trials using the antisense oligonucleotide, OGX-427, which inhibits HSP27 expression, have been performed in patients with prostate, bladder, ovarian, breast or non-small cell lung cancer, but not colorectal cancer. This therapy has been reported to be feasible and effective (26,27). The inhibition of HSP27 expression would also be clinically effective as a treatment for patients with 5-FU resistant colorectal cancer, considering the results of our previous studies (17,18).

In addition, the aim of the present study was to verify that the inhibition of HSP27 phosphorylation may also present an alternative target for the treatment of colorectal cancer to promote 5-FU sensitivity or to reduce 5-FU resistance. This study demonstrated that the stress of 5-FU exposure upregulated the phosphorylation of HSP27, particularly at the Ser78 site, in the colorectal cancer cells with high levels of HSP27 expression (HCT116 and HCT15). In the colorectal cancer cells with a low level of HSP27 expression (HT29), HSP27 expression was marginally increased, with a small amount of phosphorylation of HSP27 at the three serine residues. The phosphorylated residues of HSP27 have been reported to differ in response to different inducers (28). In these colorectal cancer cells with high levels of HSP27 expression, Ser78 was identified as the phosphorylated residue in the major phosphorylated HSP27 form induced by 5-FU stimulation.

SB203580, a p38 MAPK selective inhibitor, clearly inhibited the phosphorylation of the Ser78 residue of HSP27 induced by 5-FU exposure. Furthermore, the inhibition of HSP27 phosphorylation by SB203580 significantly promoted the sensitivity of the HCT116 and HCT15 cells to 5-FU. This effect may be caused by a reduced anti-apoptotic effect as a result of the inactivation of HSP27. In addition, SB203580 had a minimal effect on cell growth or survival *in vitro*. This result indicates that the dephosphorylation of HSP27 via the inhibition of p38 MAPK by SB203580 exerted little toxicity in these colorectal cancer cells.

In conclusion, the present study indicated that the inhibition of HSP27 phosphorylation promotes 5-FU sensitivity in colorectal cancer cells with high levels of HSP27 expression. If the agents that specifically inhibit HSP27 phosphorylation exhibit minimal toxicity, such agents may present a novel strategy for the treatment of colorectal cancer when combined with current chemotherapy using 5-FU. Further investigations into HSP27 regulation are important and are required for the development of novel treatments targeting HSP27 in colorectal cancer.
